# Visfatin exerts an anti-proliferative and pro-apoptotic effect in the human placenta cells^†^

**DOI:** 10.1093/biolre/ioae168

**Published:** 2024-11-19

**Authors:** Monika Dawid, Karolina Pich, Natalia Respekta-Długosz, Wiktoria Gieras, Małgorzata Opydo, Tomasz Milewicz, Pascal Froment, Joëlle Dupont, Agnieszka Rak

**Affiliations:** Laboratory of Physiology and Toxicology of Reproduction, Institute of Zoology and Biomedical Research, Faculty of Biology, Jagiellonian University in Krakow, Poland; Doctoral School of Exact and Natural Sciences, Jagiellonian University in Krakow, Poland; Laboratory of Physiology and Toxicology of Reproduction, Institute of Zoology and Biomedical Research, Faculty of Biology, Jagiellonian University in Krakow, Poland; Doctoral School of Exact and Natural Sciences, Jagiellonian University in Krakow, Poland; Laboratory of Physiology and Toxicology of Reproduction, Institute of Zoology and Biomedical Research, Faculty of Biology, Jagiellonian University in Krakow, Poland; Doctoral School of Exact and Natural Sciences, Jagiellonian University in Krakow, Poland; Laboratory of Physiology and Toxicology of Reproduction, Institute of Zoology and Biomedical Research, Faculty of Biology, Jagiellonian University in Krakow, Poland; Laboratory of Experimental Hematology, Institute of Zoology and Biomedical Research, Faculty of Biology, Jagiellonian University in Krakow, Poland; Department of Gynecological Endocrinology, Faculty of Medicine, Jagiellonian University Medical College, Poland; INRAE, Unité Physiologie de la Reproduction et des Comportements, France; INRAE, Unité Physiologie de la Reproduction et des Comportements, France; Laboratory of Physiology and Toxicology of Reproduction, Institute of Zoology and Biomedical Research, Faculty of Biology, Jagiellonian University in Krakow, Poland

**Keywords:** visfatin, placenta, pregnancy disorders, proliferation, apoptosis, signaling pathways

## Abstract

Visfatin regulates energy homeostasis, metabolism, inflammation, and reproduction *via* the hypothalamus-pituitary-ovary axis. Our previous study showed the visfatin gene and protein expression in the human placenta. This study aimed to investigate the *in vitro* effect of visfatin on the proliferation and apoptosis of placental JEG-3 and BeWo cells but also in villous explants collected from normal pregnancies and complicated by intrauterine growth restriction (IUGR), preeclampsia (PE), and gestational diabetes mellitus (GDM). We studied placenta cells viability, proliferation, cell cycle, proliferation/apoptotic factors and insulin receptor (INSR) expression, DNA fragmentation, CASP3/7 activity, and phosphorylation of ERK1/2, AKT, AMPKα, STAT3 with their involvement after pharmacological inhibition in visfatin action on proliferation and apoptosis. Visfatin (1, 10, 100 ng/mL) decreased the viability and proliferation of JEG-3 after 48 h, and a similar effect was observed *via* co-administration of visfatin (10 ng/mL) and insulin (10 ng/mL) in JEG-3 and BeWo after 48 h and 72 h, respectively. Visfatin reduced the transition from the G2/M phase, and expression of PCNA or cyclins D, E, A, and B in JEG-3 and PCNA in normal, IUGR, PE, and GDM placentas. It increased DNA fragmentation, CASP3/7 activity, P53, BAX/BCL2, CASP9, CASP 8, CASP3 levels in BeWo, and CASP3 expression in tested placentas. Furthermore, visfatin modulated INSR, ERK1/2, AKT, AMPKα, and STAT3 expression in JEG-3 and BeWo, and its anti-proliferative and pro-apoptotic effects occurred *via* mentioned factors. In conclusion, visfatin, by affecting the proliferation and apoptosis of human placenta cells, may be an important factor in the development and function of the organ.

## Teaser

**Summary Sentence**

Visfatin exerts an anti-proliferative and pro-apoptotic effect in the human placenta *via* insulin receptor, ERK1/2, AKT, AMPKα, and STAT3 signaling pathways.

## Graphical Abstract

**Graphical Abstract:**
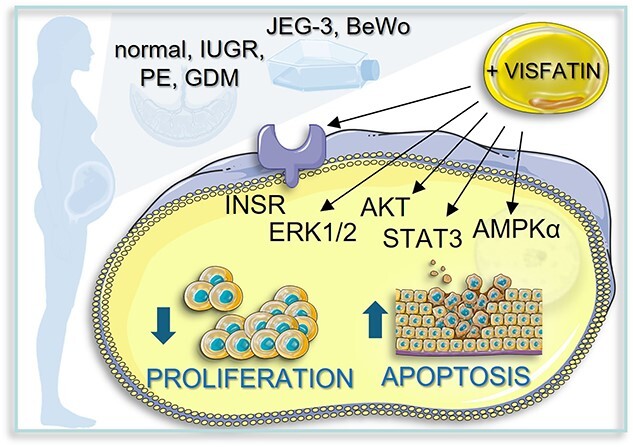

## Introduction

Pre-B cell colony enhancing factor, nicotinamide phosphoribosyltransferase (NAMPT), and visfatin refer to the same molecule, a 52-kDa protein composed of 491 amino acids in humans. This protein has several roles: as a cytokine, it can regulate inflammatory processes, as a phosphoribosyltransferase, it modulates the course of redox reactions in living cells [1]. Finally, as an adipokine, or an adipose tissue hormone, it is pleiotropic, regulating energy homeostasis, glucose metabolism, inflammation, cell differentiation, proliferation, and apoptosis [2]. Visfatin has been demonstrated to induce proliferation of breast cancer cells proliferation *via* the extracellular signal-activated kinase (ERK1/2), protein kinase B (AKT), and signal transducer and activator of transcription 3 (STAT3) pathways [3, 4]. Additionally, it has been shown to prevent interferon-γ-induced apoptosis of pancreatic islet cells through the ERK1/2 and 5′ adenosine monophosphate-activated protein kinase (AMPKα) pathways [5]. Interestingly, Fukuhara et al. [6] demonstrated that visfatin can activate the insulin (INS) receptor (INSR) and reduce glucose levels in mice. This finding has prompted ongoing debate regarding the potential role of the INSR in this process. For example, visfatin has been demonstrated to induce metalloproteinase-9 activity in human monocytes and to promote the release of the pro-inflammatory cytokines interleukin-8 (IL-8) and tumor necrosis factor-α; these effects are abolished by the blockade of INSR signaling [7]. Conversely, visfatin does not exert effects through INSR in vascular cells and macrophages [8].

In recent years, the role of visfatin in female reproduction has been studied extensively; its expression has been demonstrated in ovarian cells, where it influences the development of ovarian follicles, oocyte maturation, and steroidogenesis by affecting estrogen and progesterone secretion [9–11]. Morgan et al. [12] observed a seven-fold higher expression of visfatin in the omental fat of pregnant women compared with lean controls, and a two-times higher visfatin plasma level during pregnancy (40.3 ng/mL) compared with the control (20 ng/mL). It is also worth mentioning that visfatin levels have also been examined in pathological conditions affecting ~10% of all pregnancies. These include intrauterine growth restriction (IUGR), where the average size of the fetus is smaller than the norms adopted for a given gestational age; preeclampsia (PE), manifested by increased maternal blood pressure (≥140/90 mm Hg); or gestational diabetes mellitus (GDM), associated with a permanent hyperglycemic state of the mother [13–18]. The results regarding the maternal plasma levels of visfatin in normal and complicated pregnancies are contradictory. Mazaki-Tovi et al. [13] reported an increase in visfatin levels, whereas Birdir et al. [14] observed a decrease in the cases of IUGR. Zulfikaroglu et al. [15] noted elevated visfatin maternal plasma levels, while Hu et al. [16] documented reduced maternal serum levels in women with PE. However, Telejko et al. [17] found lower visfatin levels in women with GDM. In their meta-analysis, Jiang et al. [18] reported no significant differences between the PE group compared with the normal control. Visfatin dose-dependently protects human amniotic epithelial cells from apoptosis induced by actinomycin D *in vitro* [19]. Visfatin reduces the expression of IL-8 and tumor protein 53 (P53), thereby increasing the human amniotic cell survival *in vitro*; knocking down visfatin with antisense probes abrogates this effect [20]. Interestingly, Iciek et al. [21] showed that low visfatin expression in the placenta and poor metabolic control in the third trimester of gestation stimulate fetal overgrowth in pregnant women with diabetes. Moreover, visfatin significantly induces glucose transport in a cell culture model of the placenta [22].

Our previously published data demonstrated the expression and localization of visfatin in human placenta cell lines and placentas from normal and complicated pregnancies [23]. We observed an interesting difference related to the localization of visfatin: in placentas from pregnancies complicated by IUGR, PE, and GDM, visfatin was identified in decidual cells, while in normal placentas it was not localized in this area, which suggests the possibility of using visfatin as a diagnostic marker in pregnancy pathologies [23]. These results indicate that the variable visfatin levels observed between normal and complicated pregnancies may be related to dysregulated hormonal homeostasis, which could affect normal fetal development [23].

Normal fetal development depends on many cellular processes occurring in the placenta, including homeostasis between proliferation and apoptosis [24]. Proper proliferation maintains an appropriate pool of cells for the placenta and supports blood flow through the organ, which ensures the delivery of nutrients and oxygen to the fetus [25]. In contrast, apoptosis degrades damaged or abnormal cells, controlling the proper development of the structure and function of the placenta [26]. Abnormalities in both processes are observed in pregnancy disorders. In IUGR, there is a decrease while in PE there is an increase in proliferation [27, 28]. Additionally, both disorders show increased placental apoptosis, which translates into an impaired ability of the placenta to transport nutrients and oxygen to the fetus [26]. However, in GDM researchers have reported increased proliferation and decreased apoptosis of the placenta, which often results in fetal macrosomia [29, 30].

Cell models are often used in *in vitro* placental proliferation and apoptosis studies. Examples include choriocarcinoma cells: the BeWo cell line expresses syncytiotrophoblast-related markers such as chorionic gonadotropin and placental protein 13 [31], and the JEG-3 cell line expresses cytotrophoblast-related markers such as human leukocyte antigen G [32]. Given the higher mitotic potential of cytotrophoblasts, the JEG-3 cell line is a more suitable model for studying proliferation, whereas the BeWo cell line is more appropriate for investigating apoptosis, which is concentrated mainly in syncytiotrophoblasts [33].

We hypothesize that visfatin regulates placental function by modulating cell viability, proliferation, and apoptosis *via* different signaling pathways. The present study aimed to determine the effect of visfatin on viability, proliferation, and apoptosis along with understanding the molecular mechanisms of the observed changes using JEG-3 and BeWo placental cell lines as well as placental explants from normal, IUGR, PE, and GDM pregnancies. We determined the *in vitro* effect of visfatin on (*i*) the viability and proliferation of JEG-3 and BeWo cells; (*ii*) the cell cycle based on the expression of proliferating cell nuclear antigen (PCNA) and cyclins D, E, A, B in JEG-3 cells and placental explants from normal and complicated pregnancies; (*iii*) DNA fragmentation and enzymatic activity of caspase-3/7 (CASP3/7) in BeWo cells; (*iv*) expression of the apoptotic factors P53, CASP8, CASP9, and CASP3 as well as the Bcl-2-like protein 4/B-cell lymphoma 2 (BAX/BCL2) ratio in BeWo cells and placental explants from normal and complicated pregnancies; and (*v*) the expression and localization of INSR, phosphorylation of signaling proteins (ERK1/2, AKT, STAT3, AMPKα), and their involvement in the action of visfatin in JEG-3 and BeWo cells. The abbreviations used in the paper are listed in Supplementary Table 1.

## Materials and methods

### Ethics statement

Term placentas (38–40 weeks of gestation) from normal and pathological pregnancies (IUGR, PE, and GDM) were obtained after natural childbirth from the Clinical Department of Gynecological Endocrinology, Jagiellonian University Hospital, Krakow. Prior to the study, approval was granted by the Bioethics Committee (no: 1072.6120.252.2022). To be included in the study, patients had to meet the following criteria: a diagnosis of physiological pregnancy or IUGR or PE or GDM, informed consent to participate in the study, and an age between 20 and 40 years. Patients were excluded from participation in the study if they were not pregnant, did not consent to participate, had cancer, dyslipidemia, acute inflammatory processes, psychiatric diseases, type I or type II diabetes, or liver, kidney, or blood clotting disorders. In addition, the study did not include patients with a history of surgical treatment of ovarian lesions, chemotherapy for oncological indications, the use of drugs used in oncological chemotherapy for other indications, radiotherapy for oncological indications, irradiation for non-oncological indications, immunosuppressive treatment, patients with a history of treatment or currently treated with systemic steroids and patients treated in the ``oncofertility'' fertility preservation procedure.

### Reagents

Dulbecco’s Modified Eagle Medium/Nutrient Mixture F-12 (DMEM/F12, cat. 21,041,025), phosphate-buffered saline (PBS, cat. 14190250), alamarBlue (cat. DAL1100), the TaqMan Gene Expression Cells-to-CT Kit (cat. AM1728), and trypsin (cat. 26616) were obtained from Thermo Fisher Scientific (Waltham, MA, USA). L-glutamine (cat. G7513), INS (cat. 15523), PD098059 (cat. 1213), AG490 (cat. T3434), compound C (cat. P5499), Laemmli buffer (cat. 38733), Immobilon Western Chemiluminescent Horseradish Peroxidase (HRP) Substrate (cat. WBKLS0500), polyvinylidene fluoride (PVDF) membranes (cat. IPVH00010), and ethanol (cat. 1.00983) were purchased from Sigma-Aldrich (Saint Louis, MO, USA). Tris, glycine, Triton X-100, Tween 20 (cat. TWN510.500), and bovine serum albumin (BSA, cat. ALB001.500) were obtained from BioShop (Burlington, Canada). The Bromodeoxyuridine (BrdU) Assay Kit (cat. 11647229001) and the Cell Death Detection enzyme-linked immunosorbent assay (ELISA) Kit (cat. 11544675001) were procured from Roche Diagnostics (Basel, Switzerland). Fetal bovine serum (FBS, cat. F9665) was from BioWest (Nuaillé, France). Visfatin (cat. 8424-VF-050) was obtained from Bio-Techne (Minneapolis, MN, USA). S961 (cat. 051-86) was purchased from TargetMol Chemicals (Wellesley Hills, MA, USA). LY290042 (cat. 9901) was procured from Cell Signaling Technology (Danvers, MA, USA). Propidium iodide (PI)/RNase staining buffer (cat. 550825) was ordered from Biosciences (San Jose, CA, USA). Finally, the Caspase-Glo 3/7 Reagent (cat. G8090) was from Promega (Madison, WI, USA).

### *In vitro* culture

JEG-3 cells (cat. HTB-36; American Type Culture Collection, Manassas, VA, USA) were cultured in DMEM/F12 without phenol red, supplemented with 10% FBS. BeWo cells (cat. CCL-98; American Type Culture Collection) were cultured in DMEM/F12 with 10% FBS and 1% L-glutamine, as previously described [34]. Cells were seeded at different densities depending on the experiment: 4 × 10^3^ cells/well in 96-well plates (for AlamarBlue, BrdU, RT-qPCR, Western blotting, Cell Death Detection ELISA, and Caspase-Glo 3/7 assay), or 5 × 10^4^ cells/well in 12-well plates (for flow cytometry analysis), or 3 × 10^4^ cells/well in 8-well Lab-Tek chamber slides (for immunofluorescence). Following a 24 h incubation period, the culture medium was replaced with DMEM/F12 containing 1% FBS (JEG-3) or 10% FBS (BeWo), and the reagents were added at the specified concentrations.

**Figure 1:**
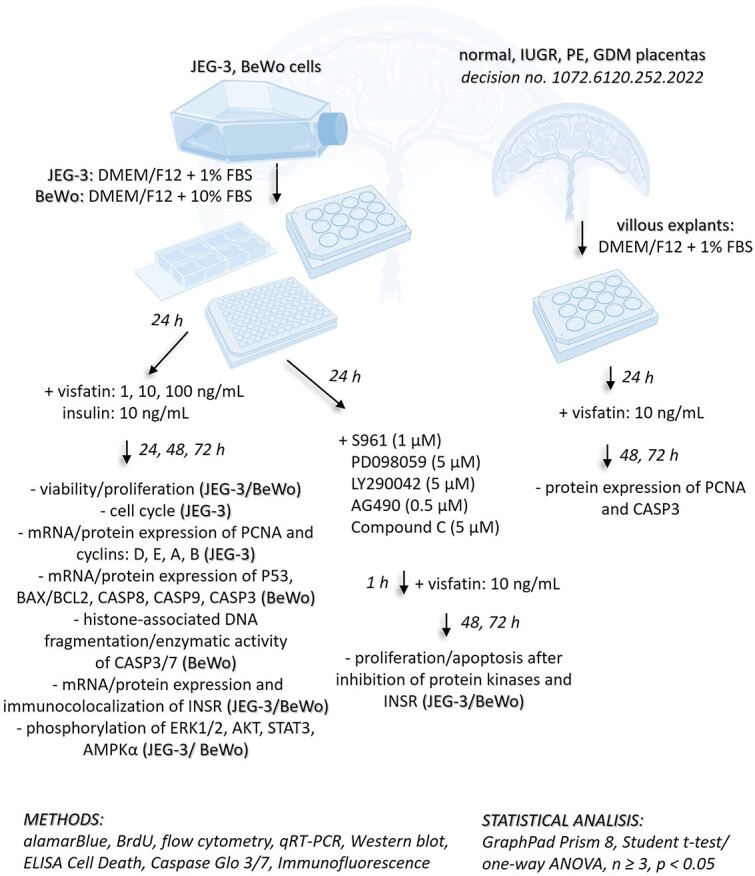
The course of the carried experiments. PCNA- proliferating cell nuclear antigen, P53- tumor protein P53, BAX- bcl-2-like protein 4, BCL2- B-cell lymphoma 2, CASP’s- caspases, INSR- insulin receptor, ERK1/2- extracellular signal-activated kinase, AKT- protein kinase B, STAT3- signal transducer and activator of transcription, AMPKα- 5'AMP-activated kinase, S961- INSR antagonist, PD098059- ERK1/2 inhibitor, LY294002- AKT inhibitor, AG490- STAT3 inhibitor, Compound C- AMPKα inhibitor.

After delivery, term placentas from normal, IUGR, PE, or GDM pregnancies were transported from the Clinical Department of Gynecological Endocrinology, University Hospital, Krakow, in PBS containing 100 IU/mL penicillin and 100 g/mL streptomycin to the laboratory. There, they were rinsed with PBS containing antibiotics. Then, placental villous explants (15 mg wet weight) were isolated and cultured in DMEM/F12 with 10% FBS in 12-well plates [35]. After 24 h, explants were treated with visfatin (10 ng/mL) dissolved in DMEM/F12 with 1% FBS as described below for Western blotting. Both cell lines and placental explants were incubated at 37°C with 5% CO_2_ and 95% humidity. Figure 1 describes the experimental protocol performed in this study.

### AlamarBlue

JEG-3 and BeWo cells were treated with visfatin (at concentrations of 1, 10, or 100 ng/mL) or INS (at a concentration of 10 ng/mL) for 24-72 h to study cell viability using alamarBlue. Further, JEG-3 was incubated for 1 h with an INSR antagonist (S961, 1 μM), or pharmacological inhibitors of ERK1/2 (PD098059, 5 μM), AKT (LY290042, 5 μM), AMPKα (Compound C, 5 μM), and STAT3 (AG490, 0.5 μM) and then visfatin (10 ng/mL) was added for 48 h to study the molecular mechanism of cells proliferation. The visfatin and INS doses were chosen based on our previous research by Mlyczyńska et al. [10] and literature data on visfatin serum levels in pregnant women [22]. The doses of the INSR antagonist and kinase inhibitors were based on the methodology employed in our previous study. AlamarBlue assay measures cell metabolic activity and viability. The reaction is based on the transformation of blue, non-fluorescent resazurin into pink, fluorescent resorufin [36]. After incubation with the reagent for 3 h (37°C, 5% CO_2_, and 95% humidity), fluorescence was measured at 570 and 600 nm using a FLUORO reader (BioTek Instruments, Winooski, VT, USA).

### BrdU assay

JEG-3 and BeWo cells were cultured with visfatin (at concentrations of 1, 10, or 100 ng/mL) or INS (at a concentration of 10 ng/mL) for a period of 48-72 h to study cell proliferation using BrdU assay. During the assay, BrdU is incorporated into newly synthesized DNA in the S phase of the cell cycle [36]. After incubation with BrdU labelling solution for 3 h (37°C, 5% CO_2_, and 95% humidity), the cells were fixed for 30 min at room temperature. Then, they were incubated with a specific anti-BrdU antibody (1.5 h at room temperature) to detect actively proliferating cells. The cells were washed three times with PBS, and then the substrate solution was added (30 min at room temperature). After incubation for 5 min with the reagent, absorbance was measured at 370 nm with a Varioskan LUX multimode microplate reader (ThermoFisher Scientific).

### RT-qPCR

JEG-3 cells and BeWo were treated with visfatin (10 ng/mL) for 24-72 h to evaluate the mRNA expression of proliferation factors in JEG-3, apoptotic factors in BeWo, and *INSR* in both cell lines. In the next step, BeWo cells were cultured for 1 h with a pharmacological antagonist of INSR (S961, 1 μM), or pharmacological inhibitors of ERK1/2 (PD098059, 5 μM), AKT (LY290042 5 μM), AMPKα (Compound C, 5 μM), and STAT3 (AG490, 0.5 μM), then visfatin (10 ng/mL) was added for 72 h to check the mRNA expression of *CASP3*. After incubation, cells were stored at −70°C for RT-qPCR. RNA was isolated from JEG-3 and BeWo cells and reverse transcribed (1 h at 37°C) using the TaqMan Gene Expression Cells-to-CT kit following the manufacturer’s protocol. The complementary DNA (cDNA) was diluted (1:5) in molecular biology-grade water and analyzed by the StepOnePlus Real-Time PCR System (Thermo Fisher Scientific). Each reaction comprised 0.5 μL of the TaqMan assay (Supplementary Table 2), 5 μL of TaqMan Gene Expression Master Mix, 2.5 μL of water, and 2 μL of cDNA (100 ng). The thermal cycling protocol was 50°C for 2 min; 95°C for 10 min; and 40 cycles of 95°C for 15 s and 60°C for 60 s. Expression of glyceraldehyde 3-phosphate dehydrogenase (*GAPDH*) was used for normalization (Supplementary Table 2). The 2^-ΔΔCt^ method was used to determine gene expression [37].

### Western blotting

JEG-3, BeWo, and placental villous explants were cultured with visfatin (10 ng/mL) for 48-72 h, and stored at −20°C for subsequent Western blotting analysis. This was conducted to study the protein expression of PCNA and cyclin D, E, A, and B, INSR in JEG-3, and P53, BAX/BCL2, CASP8, CASP9, CASP3, and INSR in BeWo, as well as PCNA and CASP3 in placental villous explants. In the further experiment, JEG-3 and BeWo cells were incubated with visfatin (10 ng/mL) for 1, 5, 10, 15, 30, 45, or 60 min to check the phosphorylated (p) and (t) total levels of ERK1/2, AKT, AMPKα, and STAT3. The protocol was the same as we described previously [38], samples (~20-40 μg of protein/lane) were loaded onto hand-cast 8%-10% polyacrylamide gels. Then, proteins were separated electrophoretically and transferred to a PVDF membrane using Trans-Blotting Turbo Mini PVDF Transfer Packs (Bio-Rad Laboratories, Hercules, CA, USA). The membranes were incubated in 0.02 M Tris-buffered saline with 5% BSA and 0.1% Tween-20 at room temperature for 1 h to block non-specific protein binding. The membranes were incubated overnight at 4°C with the appropriate primary antibodies (Supplementary Table 3). After this overnight incubation, the membranes were washed with Tris-buffered saline containing 0.1% Tween-20 and incubated with the appropriate secondary antibodies coupled to HRP (anti-rabbit or anti-mouse; Supplementary Table 3) for 1 h at room temperature. An HRP substrate was added and the chemiluminescent signal was detected using the Chemidoc XRS+ system (BioRad Laboratories). The protein bands were quantified by densitometry using ImageJ software (version 1.51, National Institutes of Health, Bethesda, MD, USA), with β-actin (ACTB) as a loading control.

### Flow cytometry

JEG-3 cells were cultured with visfatin (10 ng/mL) for 48–72 h. Then, cells were fixed with cold 70% ethanol at 4°C for 60 min, and stored at −20°C to study the cell cycle by flow cytometry as described previously [34]. JEG-3 cells were washed twice in 1 mL of PBS. The cell pellet was resuspended in 300 μL of PI/RNase staining buffer, incubated for 30 min in the dark at room temperature, and stained with PI, then fluorescence was measured with a FACS Calibur flow cytometer (Becton, Dickinson, and Company, Franklin Lakes, NJ, USA). A total of 1 × 10^3^ cells were studied per sample. The percentage of the cell population in the G0/G1, S, and G2/M phases of the cell cycle was calculated from DNA content histograms using the WinMDI 2.8 Software (The Scripps Research Institute, La Jolla, CA, USA).

### Immunofluorescence

To study localization and co-localization of visfatin and INSR, JEG-3, and BeWo cells were incubated for 48 h in DMEM/F12 with 1% or 10% FBS, respectively. After fixation with 4% paraformaldehyde for 10 min at room temperature, JEG-3 and BeWo cells were rinsed with PBS, and then incubated for 15 min in 0.1 M glycine/PBS. Cells were permeabilized with 0.15% Triton X-100 in PBS containing 1% BSA for 15 min, and incubated in 2% BSA/PBS for 15 min to block non-specific protein binding. Next, the cells were incubated with primary antibodies (anti-visfatin and anti-INSR; Supplementary Table 3) dissolved in 1% BSA/PBS for 1 h. After this incubation, the cells were washed and then incubated with anti-rabbit secondary antibodies conjugated to Alexa Fluor 488 (green fluorescence) or Alexa Fluor 594 (red fluorescence; Supplementary Table 2) for 1 h at room temperature in the dark. The negative control comprised cells for which the primary antibody was either omitted or replaced with immunoglobulin G. Examination of the cells was conducted using an Axioplan Zeiss fluorescence microscope (Boston Industries, Boston, MA, USA).

### Cell death detection ELISA

BeWo cells were incubated with visfatin (10 ng/mL) for 24-72 h, and then collected and stored at −70°C to check DNA fragmentation with the Cell Death Detection ELISA. This sandwich ELISA provides a quantitative *in vitro* measure of cytoplasmic histone-associated DNA fragments (mono- and oligonucleosomes) [39]. The absorbance was measured at 405 nm using a Varioskan LUX multimode microplate reader (ThermoFisher Scientific).

### Caspase-Glo 3/7 assay

BeWo cells were incubated with visfatin (at concentrations of 1, 10, or 100 ng/mL) alone or treated with visfatin (10 ng/mL) and INS (10 ng/mL) for 72 h to evaluate CASP3/7 activity with the Caspase Glo 3/7 test. The addition of the Caspase-Glo 3/7 reagent lyses cells, followed by CASP cleavage of the substrate. The substrate releases aminoluciferin that is consumed by luciferase to generate a luminescent signal proportional to the activity of CASP3/7. The reagent was added proportionally to the well content (1:1). After incubation for 1.5 h, luminescence was measured using a Varioskan LUX multimode microplate reader (ThermoFisher Scientific).

### Statistical analysis

All results are presented as mean ± standard error of the mean of three (term placentas) or five (cell lines) replicates. To minimize differences between the groups, the selection of cell line passages and patients for the study was carried out randomly. In addition, to control for potential confounding variables between patients, previously described inclusion and exclusion criteria were used, and additionally, all patients were characterized by normal body weight (body mass index <25 kg/m2). The reduced number of experimental repetitions with patients in comparison to cell lines was due to ethical aspects, material availability, and difficulties related to the recruitment of pregnant women, leading to the extension of the research time and its cost. However, according to previous data, a limited number of repetitions is acceptable for placental studies [40, 41]. The normality of the data distribution was assessed using the Shapiro–Wilk test. Subsequently, the data were analyzed using Student’s t-test, one-way analysis of variance (ANOVA), and Tukey’s honestly significant difference (HSD) multiple range test in GraphPad Prism 8 (GraphPad Software, San Diego, CA, USA). The Student's t-test helped to compare the means between the two independent groups to assess whether the differences between them were statistically significant under the assumption of the normality of the data distribution. A one-way ANOVA was used to compare means in more than two groups to minimize the risk of type I errors that could occur with repeated use of t-tests. Subsequently, Tukey's post-hoc test was conducted after one-way ANOVA to identify which groups were statistically significantly different. In the figures, statistical significance is indicated by letters (*P <* 0.05), where a < b < c < d < e < f < g; identical letters mean no difference. Asterisks in the tables indicate statistical significance: ^*^P < 0.05, ^*^^*^P < 0.01, and ^*^^*^^*^P < 0.001; ns indicates insignificant.

## Results

### The effect of visfatin on JEG-3 and BeWo cell viability and proliferation

**Figure 2:**
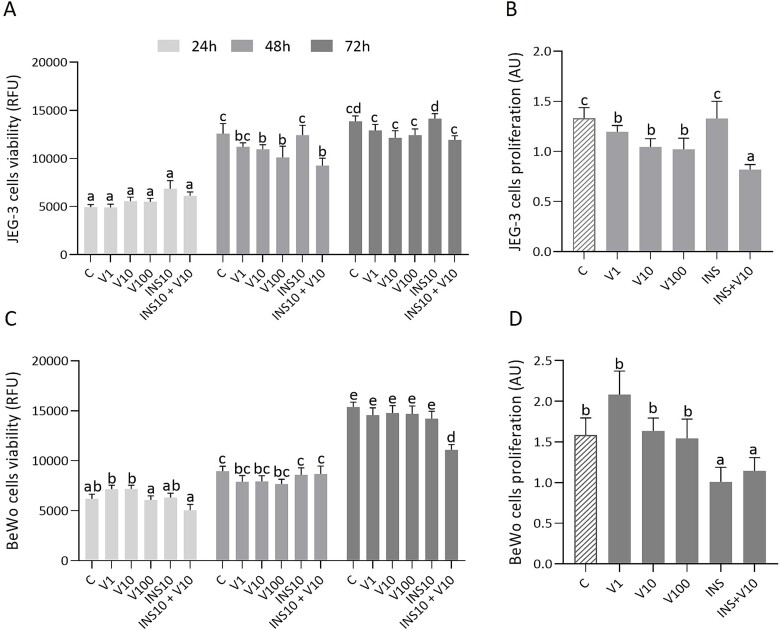
Visfatin effect at concentrations of 1, 10, 100 ng/mL on placental viability (A, C) and proliferation (B, D) in JEG-3 and BeWo cell lines. Statistical analysis was performed using a one-way ANOVA followed by Tukey’s HSD multiple range test (MEAN ± SEM, *P <* 0.05). Viability in both cell lines was studied after 24, 48, and 72 h of incubation, while proliferation in JEG-3 was examined after 48 h, and in BeWo after 72 h. C- control, V- visfatin (1, 10, 100 ng/mL), INS- insulin (10 ng/mL), RFU- relative fluorescence units, AU- arbitrary units.

Treatment for 48 h with 10 or 100 ng/mL visfatin alone or visfatin (10 ng/mL) in combination with INS (10 ng/mL) significantly decreased JEG-3 cell viability (Fig. 2A, *P <* 0.05). Treatment for 48 h with 1, 10, or 100 ng/mL or visfatin (10 ng/mL) and INS (10 ng/mL) significantly decreased JEG-3 proliferation (Figure 2B, *P <* 0.05). For BeWo cells, only the treatment for 72 h with visfatin (10 ng/mL) and INS (10 ng/mL) decreased viability (Figure 2C, *P <* 0.05). However, treatment for 72 h with INS (10 ng/mL) or visfatin (10 ng/mL) and INS (10 ng/mL) decreased BeWo cell proliferation (Figure 2D, *P <* 0.05).

### Effect of visfatin on the mRNA expression of proliferation factors in JEG-3 cells

Compared with the control, treatment for 48 or 72 h with visfatin (10 ng/mL) reduced *CCND1*, *CCNE1*, and *CCNB1* mRNA expression, while treatment for 72 h decreased *PCNA* and *CCNA2* mRNA expression after 72 h (Table 1, *P <* 0.05).

**Table 1:** Visfatin effect at a concentration of 10 ng/mL on the mRNA expression of proliferation factors normalized to the *GAPDH* reference gene after 48 and 72 h of incubation in the JEG-3 cell line. Statistical analysis was performed using Student’s t-test (MEAN ± SEM, ^*^P < 0.05, ^*^^*^P < 0.01, ^*^^*^^*^P < 0.001). C- control, V10- visfatin (10 ng/mL), *PCNA*- proliferating cell nuclear antigen, *CCND1*- cyclin D, *CCNE1*- cyclin E, *CCNA2*- cyclin A, *CCNB*- cyclin B1, *GAPDH*- glyceraldehyde 3-phosphate dehydrogenase, ns- no significant effect

	48 h			72 h		
Genes	C	V10	*P <* 0.05	C	V10	*P <* 0.05
*PCNA*	1.02 (±0.02)	1.01 (±0.11)	ns	1.22 (±0.15)	1.02 (±0.11)	^*^^*^^*^
*CCND1*	1.03 (±0.19)	0.88 (±0.07)	^*^	1.02 (±0.04)	0.81 (±0.06)	^*^^*^^*^
*CCNE1*	1.00 (±0.02)	0.92 (±0.06)	^*^^*^	1.04 (±0.07)	0.86 (±0.06)	^*^^*^^*^
*CCNA2*	1.14 (±0.50)	0.91 (±0.11)	ns	1.03 (±0.11)	0.80 (±0.11)	^*^^*^
*CCNB1*	1.02 (±0.14)	0.90 (±0.04)	^*^	0.99 (±0.07)	0.88 (±0.03)	^*^^*^^*^

### The effect of visfatin on the protein expression of cell cycle and proliferation factors in JEG-3 cells and placental villous explants from normal and complicated pregnancies

**Figure 3:**
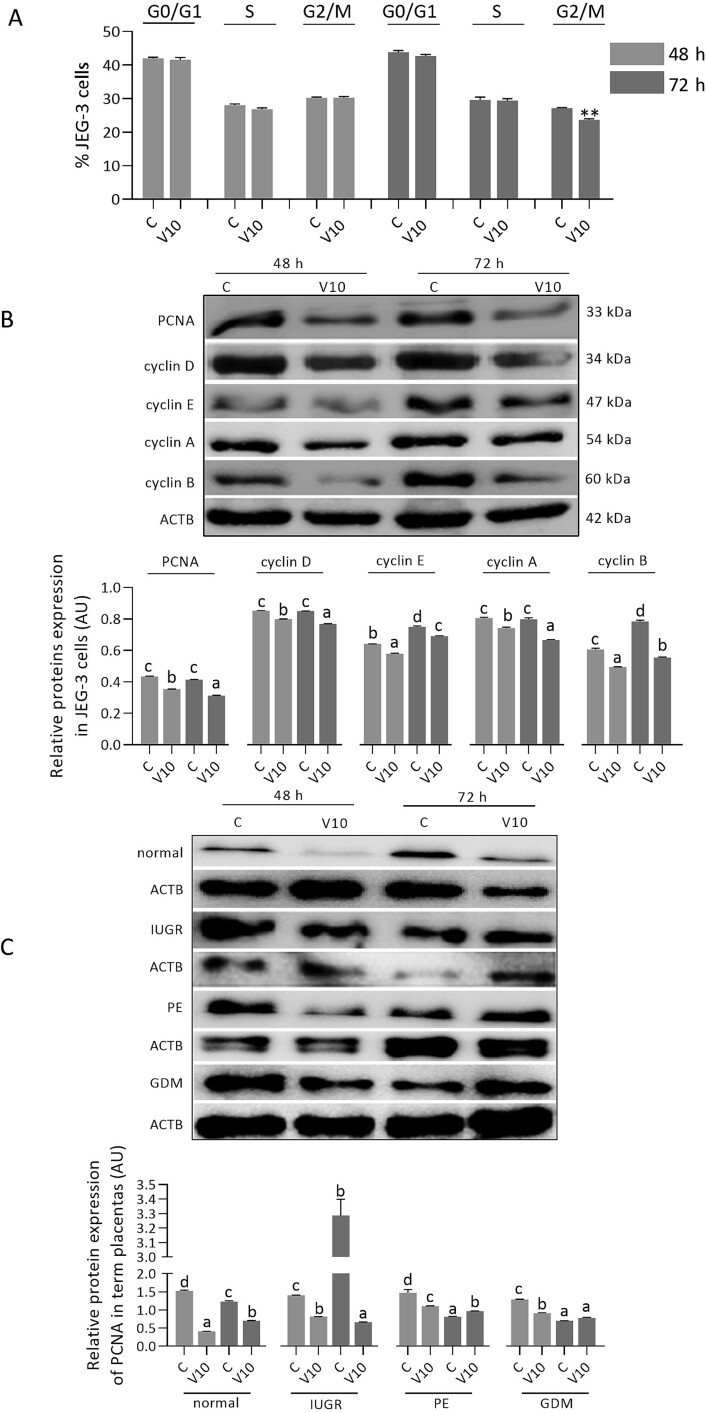
Visfatin effect at a concentration of 10 ng/mL on the cell cycle (A) and protein expression of PCNA and cyclins D, E, A, and B in JEG-3 cells (B) and placental explants (C) from normal and pathological pregnancies normalized to ACTB, after 48 and 72 h of incubation. Statistical analysis was performed using Student’s t-test (MEAN ± SEM, ^*^^*^P < 0.05) or one-way ANOVA followed by Tukey’s HSD multiple range test (MEAN ± SEM, *P <* 0.05). C- control, V10- visfatin (10 ng/mL), PCNA- proliferating cell nuclear antigen, ACTB- β-actin, IUGR- intrauterine growth restriction, PE- preeclampsia, GDM- gestational diabetes mellitus, AU- arbitrary units.

Incubation with visfatin for 72 h significantly reduced the percentage of JEG-3 cells in the G2/M phase of the cell cycle (Figure 3A, *P <* 0.05). Moreover, treatment for 48 or 72 h with visfatin (10 ng/mL) diminished PCNA and cyclin D, E, A, and B protein expression in JEG-3 cells (Figure 3B, *P <* 0.05). PCNA expression was also reduced in normal and IUGR villous explants after incubation for 48 or 72 h with visfatin (10 ng/mL). In contrast, in PE villous explants there was a significant decrease in PCNA level after 48 h and an increase after 72 h, while in GDM villous explants, visfatin reduced PCNA expression after 48 h and no changes were observed after 72 h (Figure 3C, *P <* 0.05).

### The effect of visfatin on the expression of INSR and the phosphorylation of signaling pathway components in JEG-3 cells

**Figure 4:**
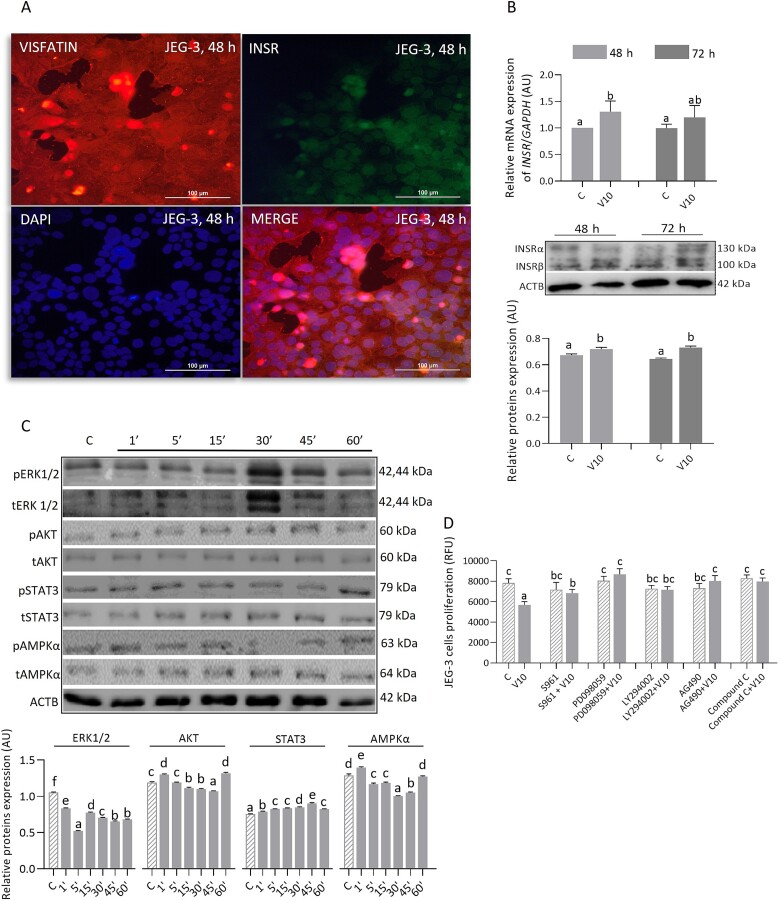
Immunolocalization of visfatin and INSR (A), visfatin effect at a concentration of 10 ng/mL on mRNA and protein expression of INSR (B), and protein kinases phosphorylation (C) along with the molecular mechanism of proliferation (D) in JEG-3 cells. Statistical analysis was performed using one-way ANOVA followed by Tukey’s HSD multiple range test (MEAN ± SEM, *P <* 0.05); image magnification × 40, scale bar 100 μm. The effect of visfatin on INSR expression was examined after 48 and 72 h of incubation, kinase phosphorylation after 1, 5, 15, 30, 45, 60 min, and molecular mechanism after 48 h. C- control, V10- visfatin (10 ng/mL), INSR- insulin receptor, ERK1/2- extracellular signal-activated kinase, AKT- protein kinase B, STAT3- signal transducer and activator of transcription 3, AMPKα- 5'AMP-activated kinase, S961- INSR antagonist, PD098059- ERK1/2 inhibitor, LY294002- AKT inhibitor, AG490- STAT3 inhibitor, Compound C- AMPKα inhibitor, ACTB- β-actin, AU- arbitrary units, RFU- relative fluorescence units.

We observed visfatin and INSR in the cytoplasm and nucleus of JEG-3 cells; these proteins co-localized in both areas (Figure 4A). Treatment for 48 or 72 h with visfatin (10 ng/mL) did not affect *INSR* mRNA expression but significantly increased INSR protein expression (Figure 4B, *P <* 0.05). At all examined time points, visfatin (10 ng/mL) reduced ERK1/2 phosphorylation but increased STAT3 phosphorylation. In addition, it had a modulatory (stimulatory or inhibitory) or no effect on AKT and AMPKα phosphorylation (Figure 4C, *P <* 0.05). The anti-proliferative effect of visfatin was eliminated after pharmacological inhibition of INSR and mentioned protein kinases, which proves the involvement of INSR and the tested signaling pathways in this process (Figure 4D, *P <* 0.05).

### The effect of visfatin on DNA fragmentation and CASP3/7 activity in BeWo cells

**Figure 5:**
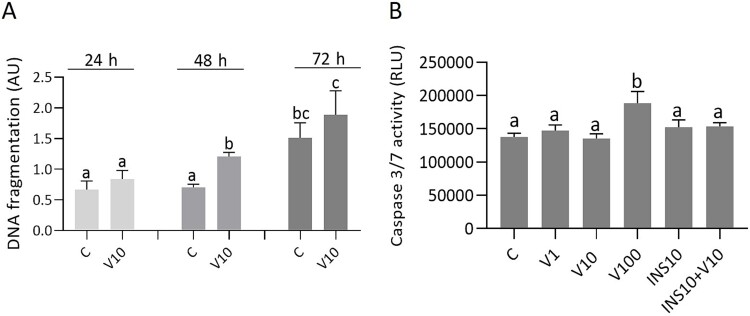
Histone-associated DNA fragmentation (A) and enzymatic activity of CASP3/7 (B) in BeWo cells after treatment with visfatin at concentrations of 1, 10, or 100 ng/mL and INS at a concentration of 10 ng/mL. Statistical analysis was performed using one-way ANOVA followed by Tukey’s HSD multiple range test (MEAN ± SEM, *P <* 0.05). Histone-associated DNA fragmentation was examined after 24, 48, and 72 h of incubation, while the enzymatic activity of CASP3/7 only after 72 h. C- control, V1, V10, V100- visfatin (1, 10, 100 ng/mL), INS- insulin, AU- arbitrary units, RLU- relative light units.

Treatment for 48 or 72 h with 10 ng/mL visfatin significantly increased DNA fragmentation compared with the control (Figure 5A, *P <* 0.05). Treatment for 72 h with 100 ng/mL visfatin increased CASP3/7 activity, while INS alone or co-administration of visfatin and INS had no effect (Figure 5B, *P <* 0.05).

### The effect of visfatin on the mRNA expression of apoptotic factors in BeWo cells

As shown in Table 2, treatment for 24, 48, or 72 h with visfatin (10 ng/mL) significantly increased *P53* and *CASP8* mRNA expression, the *BAX*/*BCL2* ratio, and *CASP8* (*P <* 0.05). Treatment for 48 or 72 h with visfatin (10 ng/mL) increased *CASP9* expression (*P <* 0.01). Finally, 24 or 72 h treatment with visfatin (10 ng/mL) increased *CASP3* expression (*P <* 0.001).

**Table 2:** Visfatin effect at a concentration of 10 ng/mL on the mRNA expression of apoptotic factors normalized to the *GAPDH* reference gene after 24, 48, and 72 h of incubation in the BeWo cell line. Statistical analysis was performed using Student’s t-test (MEAN ± SEM, ^*^P < 0.05, ^*^^*^P < 0.01, ^*^^*^^*^P < 0.001). C- control, V10- visfatin (10 ng/mL), *P53*- tumor protein 53, *BAX*- bcl-2-like protein 4, *BCL2*- B-cell lymphoma 2, *CASP8*- caspase 8, *CASP9*- caspase 9, *CASP3*- caspase 3, *GAPDH*- glyceraldehyde 3-phosphate dehydrogenase, ns- no significant effect

	24 h			48 h			72 h		
Genes	C	V10	*P <* 0.05	C	V10	*P <* 0.05	C	V10	*P <* 0.05
*P53*	0.94 (±0.11)	1.34 (±0.41)	^*^	1.03 (±0.29)	1.49 (±0.28)	^*^^*^	1.14 (±0.49)	2.09 (±0.46)	^*^^*^^*^
*BAX/BCL2*	0.73 (±0.18)	1.02 (±0.01)	^*^^*^^*^	1.06 (±0.04)	1.22 (±0.13)	^*^^*^^*^	0.86 (±0.12)	1.13 (±0.13)	^*^^*^^*^
*CASP8*	0.84 (±0.14)	1.17 (±0.19)	^*^^*^^*^	0.88 (±0.31)	1.69 (±0.44)	^*^^*^^*^	0.76 (±0.11)	1.48 (±0.73)	^*^
*CASP9*	0.90 (±0.18)	1.99 (±0.30)	ns	1.24 (±0.35)	1.72 (±0.25)	^*^^*^^*^	1.37 (±0.06)	1.92 (±0.13)	^*^^*^^*^
*CASP3*	0.90 (±0.11)	1.28 (±0.45)	^*^^*^	1.30 (±0.25)	1.39 (±0.21)	ns	1.37 (±0.59)	1.92 (±0.52)	^*^^*^

### The effect of visfatin on the protein expression of apoptotic factors in BeWo cells and placental villous explants from normal and complicated pregnancies

**Figure 6:**
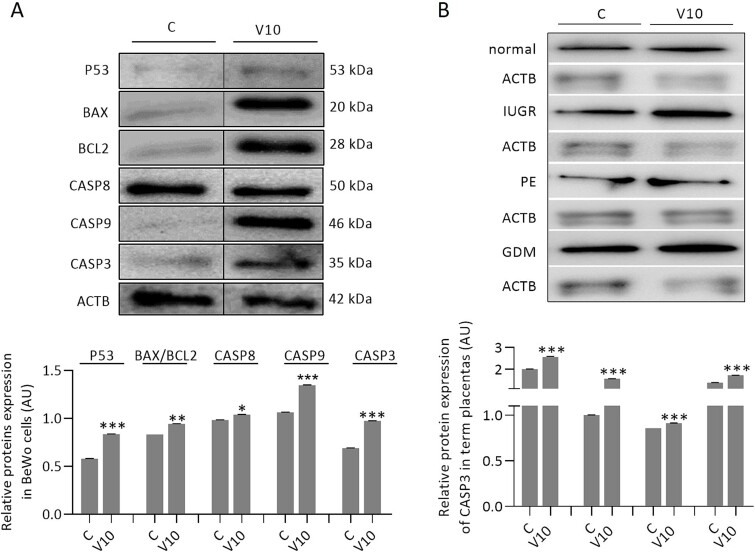
Visfatin effect at a concentration of 10 ng/mL on protein expression of apoptotic factors in BeWo cells (A) and placental explants (B) normalized to ACTB after 72 h of incubation. Statistical analysis was performed using Student’s t-test (MEAN ± SEM, ^*^P < 0.05, ^*^^*^P < 0.01, ^*^^*^^*^P < 0.001*)*. C- control, V10- visfatin (10 ng/mL), P53- Tumor protein P53, BAX- bcl-2-like protein 4, BCL2- B-cell lymphoma 2, CASP8- caspase 8, CASP9- caspase 9, CASP3- caspase 3, ACTB- β-actin, IUGR- intrauterine growth restriction, PE- preeclampsia, GDM- gestational diabetes mellitus, AU- arbitrary units.

We noted that treatment for 72 h of BeWo cells with visfatin (10 ng/mL) significantly increased P53, CASP8, CASP9, and CASP3 protein expression, and the BAX/BCL2 ratio (Figure 6A, *P <* 0.05). Treatment for 72 h with visfatin (10 ng/mL) also increased CASP3 protein expression in normal villous explants and complicated pregnancies IUGR, PE, and GDM compared to control (Figure 6B, *P <* 0.05).

### The effect of visfatin on the expression of INSR and phosphorylation of signaling pathway components in BeWo cells

**Figure 7:**
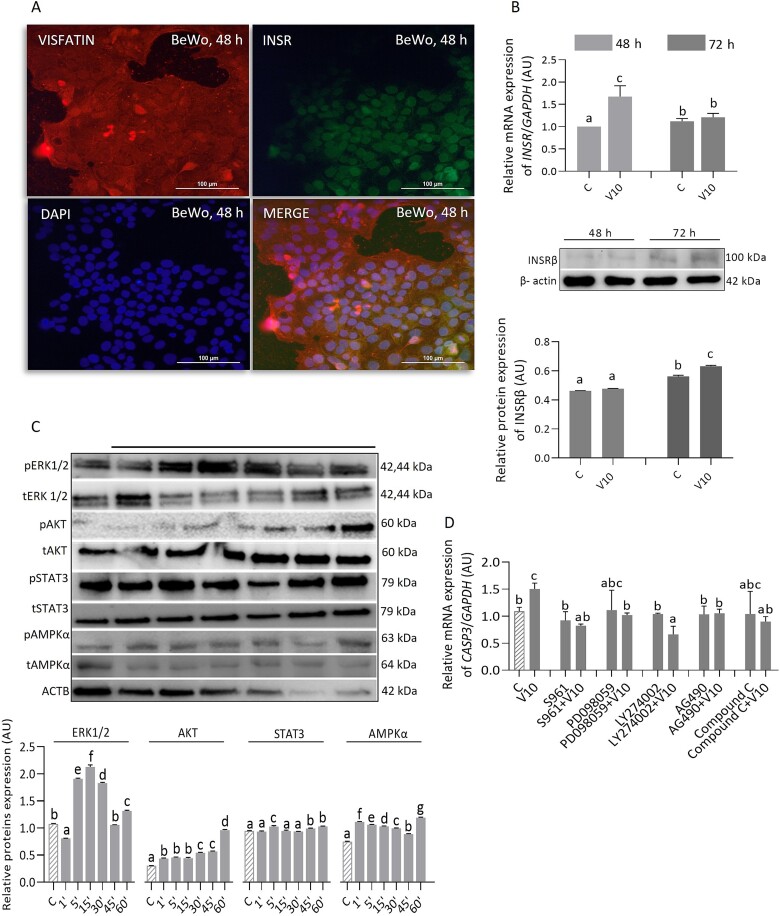
Immunolocalization of visfatin and INSR (A), visfatin effect at a concentration of 10 ng/mL on mRNA and protein expression of INSR (B), and protein kinases phosphorylation (C) along with molecular mechanism of apoptosis (D) in BeWo cells. Statistical analysis was performed using one-way ANOVA followed by Tukey’s HSD multiple range test (MEAN ± SEM, *P <* 0.05); image magnification × 40, scale bar 100 μm. The effect of visfatin on INSR expression was examined after 48 and 72 h of incubation, kinase phosphorylation after 1, 5, 15, 30, 45, 60 min, and molecular mechanism after 72 h. C- control, V10- visfatin (10 ng/mL), INSR- insulin receptor, ERK1/2- extracellular signal-activated kinase, AKT- protein kinase B, STAT3- signal transducer and activator of transcription 3, AMPKα- 5'AMP-activated kinase, ACTB- β-actin, S961- INSR antagonist, PD098059- ERK1/2 inhibitor, LY294002- AKT inhibitor, AG490- STAT3 inhibitor, Compound C- AMPKα inhibitor, AU- arbitrary units, RFU- relative fluorescence units.

Similarly to JEG-3 cells, we observed visfatin and INSR in the cytoplasm and nucleus of BeWo cells, and these proteins co-localized in both areas (Figure 7A). Treatment for 48 h with visfatin (10 ng/mL) increased *INSR* mRNA expression, while treatment for 72 h increased INSR protein expression (Figure 7B, *P <* 0.05). Furthermore, visfatin (10 ng/mL) increased ERK1/2, STAT3, and AMPKα phosphorylation after incubation for 5 min, and AKT phosphorylation at each tested time point (Figure 7C, *P <* 0.05). After pharmacological inhibition of INSR and mentioned protein kinases, the pro-apoptotic effect of visfatin, demonstrated by increased *CASP3* mRNA expression, was eliminated, implicating the tested signaling pathways in the process (Figure 7D, *P <* 0.05).

**Figure 8:**
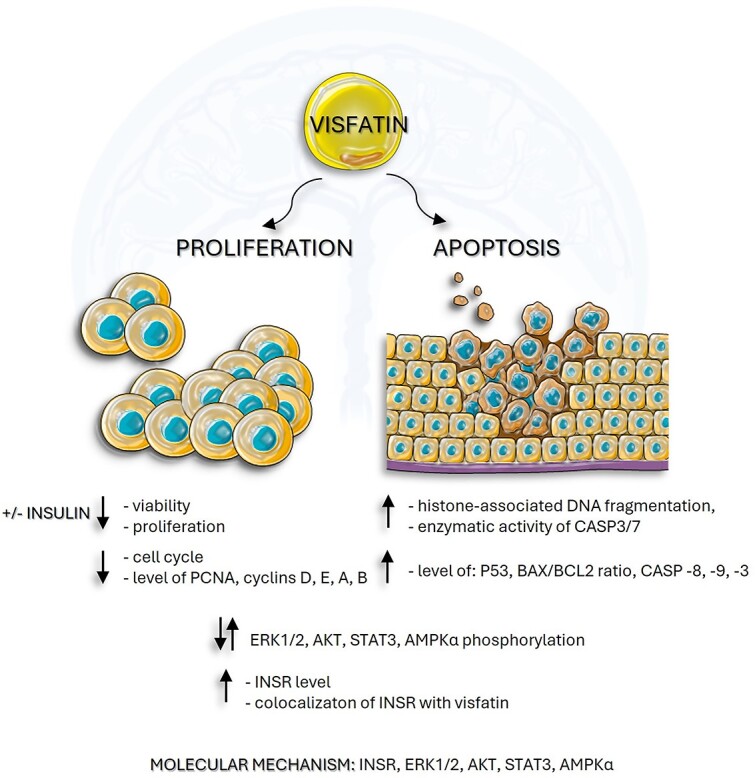
Summary of the obtained results. PCNA- proliferating cell nuclear antigen, P53- tumor protein P53, BAX- bcl-2-like protein 4, BCL2- B-cell lymphoma 2, CASP’s- caspases, ERK1/2- extracellular signal-activated kinase, AKT- protein kinase B, STAT3- signal transducer and activator of transcription 3, AMPKα- 5'AMP-activated kinase.

## Discussion

This is the first report to demonstrate that visfatin when administered alone or in combination with INS, has an inhibitory effect on the viability and proliferation of JEG-3 cells. Furthermore, the co-administration of visfatin and INS resulted in a comparable impact on BeWo cells. Additionally, visfatin reduced the percentage of JEG-3 cells in the G2/M phase of the cell cycle and the expression of PCNA and cyclins D, E, A, and B in JEG-3 cells. Furthermore, the expression of PCNA was found to be reduced in normal, IUGR, PE, and GDM villous explants. Moreover, visfatin enhanced DNA fragmentation, CASP3/7 activity, P53, CASP9, CASP8, and CASP3 expression, and the BAX/BCL2 ratio in BeWo cells, and increased CASP3 expression in normal, IUGR, PE, and GDM villous explants. Visfatin modulated the expression of INSR, ERK1/2, AKT, AMPKα, and STAT3 in JEG-3 and BeWo cells. Pharmacological blockade of INSR and these kinases reversed the anti-proliferative and pro-apoptotic effects of visfatin. These findings indicate that these pathways are involved in the molecular action of visfatin on placental proliferation and apoptosis.

The initial proliferation experiments were conducted on two placental cell lines. The JEG-3 cell line demonstrated a reduction in viability and proliferation when exposed to visfatin alone or in combination with INS. Similarly, the BeWo cell line exhibited comparable outcomes when treated with a combination of visfatin and INS. Despite the fact that both cell lines were derived from a malignant tumor that developed from trophoblasts in the placental tissue (choriocarcinoma), they may exhibit disparate responses to the tested substances. The potential for differences may be attributed to several biological or technical factors, including differentiation (different degrees of differentiation towards trophoblasts), genetic changes (e.g., resulting from the long-term maintenance of the culture *in vitro*), differential gene expression (with an impact on signaling pathways), or even the culture environment (composition of the medium, pH, and oxygen concentration) [42–45]. Based on microarray analysis, Burleigh et al. [46] reported that ~2700 genes are differentially expressed between JEG-3 and BeWo cells, which proves their pivotal and occasionally exclusive role in placental process studies. Furthermore, our findings align with previous studies that have demonstrated the suitability of JEG-3 cells as a good model for studying proliferation [47, 48]. In addition, the effect of INS on proliferation may depend on numerous factors, including cell type, concentration, exposure time, and the presence of other hormones [49, 50]. For example, INS has been demonstrated to increase the seeding and proliferation of human-induced pluripotent stem cells. Conversely, the lack of the hormone has been shown to affect changes in the cell cycle profile and increase apoptosis [51]. Nevertheless, research has indicated that INS may inhibit the proliferation of placental cells or reduce their number in certain circumstances, which is consistent with our findings. One study has confirmed that INS exerts an anti-proliferative and hypertrophic effect on HTR8/SVneo cells, a first-trimester extravillous human trophoblast cell line [52]. Furthermore, elevated levels of INS have been demonstrated to exert a toxic effect on first-trimester trophoblasts, leading to DNA damage and apoptosis, and consequently reducing cell survival [53].

Visfatin was observed to inhibit JEG-3 cell cycle progression and reduce the expression of PCNA and cyclins D, E, A, and B. These findings are inconsistent with the pro-proliferative role of visfatin in malignant melanoma (Me45), human hepatoma (HepG2, Hep3B, HuH7), breast cancer (MCF-7, MDA-MB-231), and endometrial cancer (Ishikawa, KLE) [54]. Nevertheless, the inhibition of visfatin has been observed to enhance ovarian proliferation, as evidenced by elevated PCNA expression and BrdU incorporation [55]. It can therefore be surmised that the action of visfatin is dependent on the organ in question, and based on the results obtained from the JEG-3 and BeWo cells, this dependency is also reliant on the cell type of a given organ.

As previously stated, the JEG-3 cell line was derived from choriocarcinoma. It is essential to acknowledge the model's inherent limitations and validate the results obtained. Consequently, an evaluation was also conducted on villous explants derived from the term placentas. Furthermore, the utilization of human tissues is corroborated by our preceding, unpublished findings, which illustrate the expression and localization of visfatin in discrete compartments of the fetal and maternal regions of the placenta from both normal and complicated pregnancies [23]. What are the reasons for the importance of studying the factors that regulate placental proliferation? Proliferative disorders have been linked to a range of gestational pathologies, including IUGR, PE, and GDM. Such placental deficiencies may result from a disruption in the production of growth factors that are essential for meeting fetal needs [56]. Moreover, it has been demonstrated that cell populations within the placenta exhibit staining for a multitude of cell cycle proteins. The specific staining patterns observed depend on several factors, including the presence of placental abnormalities, which represent a significant etiological factor in the development of IUGR. This condition is characterized by impaired fetal growth, which is frequently accompanied by an inadequate placenta. The researchers observed a markedly diminished level of PCNA immunostaining in dexamethasone-induced IUGR relative to that observed in the normal placentas of Wistar rats [57]. PE is associated with vascular damage and endothelial dysfunction, which result in placental ischemia or hypoxia. The hypoxia itself has been demonstrated to reduce proliferation and induce apoptosis in the placenta [58, 59]. It has been suggested that placentas associated with PE may be linked to irregularities in cell proliferation and cell cycle arrest. In comparison to normal placentas, Unek et al. [60] observed that PE placentas exhibited markedly elevated PCNA staining in the villous regions, diminished staining in the basal plates, and no discernible change in the chorionic plates. GDM is characterized by impaired glucose metabolism, which often results in hyperinsulinemia. It has been proposed that hyperglycemia in GDM may increase the rate of glucose transport *via* the placenta, which in turn may result in greater INS production in the fetus, thereby inducing its growth [61]. Furthermore, GDM placentas demonstrate a notable elevation in PCNA expression in decidual cells, connective tissue cells, and endothelial cells [62]. In conclusion, the anti-proliferative effect of visfatin, which is achieved by reducing the level of proliferation markers such as PCNA, may play a pivotal role in the pathogenesis of the above-mentioned pathologies.

The molecular mechanisms that underlie visfatin actions involve the INSR and several kinases, namely ERK1/2, AKT, AMPKα, and STAT3. Our findings are consistent with those of previous studies which have demonstrated that visfatin can modulate the activation of the INSR, ERK1/2, AKT, and AMPKα signaling pathways in porcine anterior pituitary cells [63]. Visfatin has been demonstrated to modulate INS secretion, INSR phosphorylation, intracellular signaling, and the expression of multiple mouse β-cell function-associated genes *via* INSR and ERK1/2 [64]. Moreover, the proliferation of breast and endometrial cancer cell lines may occur *via* the ERK1/2 and AKT pathways [54]. Interestingly, we previously determined that adipokine apelin stimulates trophoblast proliferation *via* its receptor, as well as the ERK1/2, STAT3, and AMPKα signaling pathways [35].

Due to the lack of a clear answer regarding the role of visfatin in proliferation and prior reports indicating that BeWo cells are better suited to study placental apoptosis [35, 65], we used this cell line. The process of placental apoptosis is a natural phenomenon that increases as pregnancy progresses, and it is related to the gradual aging of the organ [66]. Our findings indicate that visfatin enhances histone-associated DNA fragmentation, a distinctive morphological alteration frequently observed in apoptotic cells, and CASP3/7 activity, thereby substantiating its pro-apoptotic impact within the placenta. The use of extracellular histones as a biomarker has the potential to enhance the diagnosis and prognosis of various diseases, including chronic obstructive pulmonary disease and brain damage. However, interpreting changes in histones remains a significant challenge [67]. Furthermore, the pro-apoptotic effect of visfatin was confirmed based on elevated expression of P53, CASP8, CASP9, and CASP3, as well as a higher BAX/BCL2 ratio. The activation of the initiator/effector caspase cascade and other factors involved in the external and internal apoptotic pathways is a crucial step in the commitment of a cell to this type of programmed cell death [68]. Prior research has additionally demonstrated that endogenous visfatin facilitates apoptosis in murine immune organs by regulating BCL2, BAX, and CASP3 expression [69]. However, Annie et al. [70] have reported that visfatin inhibits apoptosis by upregulating BCL2 expression, thereby preserving the quality of follicles during the early postnatal period. Furthermore, the pro-apoptotic role of visfatin was evaluated in normal, IUGR, PE, and GDM villous explants, where visfatin was observed to increase CASP3 expression. Moreover, apoptosis plays a role in the physiological mechanisms of vasculogenesis and angiogenesis, regulating the formation of the lumen of blood vessels and angiogenic branches in the placenta. Abnormal placental vascular transport may be an indirect cause of IUGR [71]. Furthermore, elevated apoptosis in pregnancies complicated by IUGR is associated with elevated CASP3 expression [72], whereas pregnant women with PE exhibit reduced expression of the anti-apoptotic protein BCL2 [73]. However, in women with GDM, Belkacemi et al. [74] observed a notable decline in apoptotic processes, accompanied by a reduction in CASP3 expression. Additionally, placentas from pregnancies complicated by GDM display pyknotic and apoptotic changes in decidual cell nuclei when compared to those from pregnancies without complications [62]. It is therefore imperative to identify the factors and mechanisms regulating placental proliferation and apoptosis in order to abrogate numerous pregnancy pathologies.

We found that visfatin modulated INSR and ERK1/2, AKT, AMPKα, and STAT3 phosphorylation. Furthermore, the pro-apoptotic effect of visfatin in BeWo cells was found to occur through these factors. An intriguing discrepancy was observed between the JEG-3 and BeWo cell lines. Western blotting analysis demonstrated that the INSRβ isoform was expressed exclusively in visfatin-treated BeWo cells, whereas both the INSRα and INSRβ isoforms were expressed in visfatin-treated JEG-3 cells. In physiological *in vivo* conditions in which INS is present, INSRα has been demonstrated to be more effective than INSRβ in increasing proliferation and inhibiting apoptosis. INSRα has been demonstrated to possess oncogenic properties, which facilitate cancer cell growth. Conversely, INSRβ has been shown to exert anti-cancer effects by limiting cell proliferation [75]. Furthermore, visfatin may exert a protective or inductive effect on apoptosis. In mouse pancreatic islet cells, visfatin has been demonstrated to decrease apoptosis *via* the ERK1/2 and AMPK pathways [5]. Nevertheless, visfatin has been demonstrated to inhibit apoptosis in RAW264.7 cells, a mouse macrophage cell line, by participating in the AKT signaling pathway [69]. Moreover, visfatin has been shown to prevent endoplasmic reticulum stress-induced apoptosis by activating the STAT3 pathway in macrophages [76]. Additionally, our previous research has demonstrated the anti-apoptotic effect of apelin in placental cells, which is regulated by the ERK1/2 and AKT signaling pathways [33]. Furthermore, leptin has been demonstrated to possess anti-apoptotic properties, which are mediated by the ERK1/2 pathway [77]. Conversely, adiponectin, in a manner analogous to visfatin, has been shown to induce apoptosis in the placenta [78].

Finally, why is it important to study proliferation and apoptosis processes in pregnancy pathologies such as IUGR, PE, or GDM? These pathologies are frequently considered together due to the shared etiology of an abnormally perfused placenta [79], and structural changes in the organ may result from an inappropriate course of proliferation and apoptosis [26, 80]. At present, there are no pharmacological treatments available for IUGR. In contrast, for PE or GDM, the mainstay of therapy is the control of risk factors, such as blood pressure and blood glucose levels. Nevertheless, there is a possibility that adipokines may be regarded as a potential medical factor in the future. Previous studies have primarily focused on examining the alterations in adipokine levels between normal and complicated pregnancies [81]. It has been demonstrated that leptin inhibits apoptosis of placental cells in PE, and due to its pro-angiogenic effect, it can enhance blood flow to the placenta [82]. Another example is adiponectin, which demonstrated a reduced expression and pro-proliferative effect in the GDM placenta when compared to the normal control group [83]. To date, there have been no reports on the molecular mechanisms by which adipokines influence cellular processes in pathological placentas.

In summary, we demonstrated the anti-proliferative and pro-apoptotic effects of visfatin on human placental cells, which suggest its significant involvement in the placenta development and functions (Figure 8). The main obstacle to further research was the unavailability of placentas from normal and complicated pregnancies, which significantly reduced the number of analyses that could be carried out. Despite our best efforts to elucidate the molecular mechanism of visfatin's action on placental proliferation and apoptosis using cell lines, it remains challenging to discuss the potential therapeutic implications in the context of pregnancy pathologies. Therefore, future studies on the *in vivo* model should concentrate on elucidating the functions of visfatin in the context of pregnancy physiology and pathology. This will facilitate an understanding of whether modulation of visfatin levels could be beneficial in both the prevention and treatment of specific pregnancy disorders.

## Supplementary Material

Supplementary_Table_1_ioae168

Supplementary_Table_2_ioae168

Supplementary_Table_3_ioae168

## Data Availability

The data underlying this article will be shared on reasonable request to the corresponding author.

## References

[1] Adeghate E . Visfatin: structure, function and relation to diabetes mellitus and other dysfunctions. Curr Med Chem 2008; 15:1851–1862. 18691043 10.2174/092986708785133004

[2] Sonoli SS, Shivprasad S, Prasad CVB, Patil AB, Desai PB, Somannavar MS. Visfatin—a review. Eur Rev Med Pharmacol Sci 2011; 15:9–14. 21381495

[3] Hung AC, Lo S, Hou MF, Lee YC, Tsai CH, Chen YY, Liu W, Su YH, Lo YH, Wang CH, Wu SC, Hsieh YC, et al. Extracellular Visfatin-promoted malignant behavior in breast cancer is mediated through c-Abl and STAT3 activation. Clin Cancer Res 2016; 22:4478–4490. 27036136 10.1158/1078-0432.CCR-15-2704

[4] Gholinejad Z, Kheiripour N, Nourbakhsh M, Ilbeigi D, Behroozfar K, Hesari Z, Golestani A, Shabani M, Einollahi N. Extracellular NAMPT/Visfatin induces proliferation through ERK1/2 and AKT and inhibits apoptosis in breast cancer cells. Peptides 2017; 92:9–15. 28442350 10.1016/j.peptides.2017.04.007

[5] Xiang RL, Mei M, Su YC, Li L, Wang JY, Wu LL. Visfatin protects rat pancreatic β-cells against IFN-γ-induced apoptosis through AMPK and ERK1/2 Signaling pathways. Biomed Environ Sci 2015; 28:169–177. 25800441 10.3967/bes2015.023

[6] Fukuhara A, Matsuda M, Nishizawa M, Segawa K, Tanaka M, Kishimoto K, Matsuki Y, Murakami M, Ichisaka T, Murakami H, Watanabe E, Takagi T, et al. Visfatin: a protein secreted by visceral fat that mimics the effects of insulin. Science 2005; 307:426–430. 15604363 10.1126/science.1097243

[7] Dahl TB, Yndestad A, Skjelland M, Øie E, Dahl A, Michelsen A, Damås JK, Tunheim SH, Ueland T, Smith C, Bendz B, Tonstad S, et al. Increased expression of Visfatin in macrophages of human unstable carotid and coronary atherosclerosis: possible role in inflammation and plaque destabilization. Circulation 2007; 115:972–980. 17283255 10.1161/CIRCULATIONAHA.106.665893

[8] Romacho T, Sánchez-Ferrer CF, Peiró C. Visfatin/Nampt: an Adipokine with cardiovascular impact. Mediators Inflamm 2013; 2013:946427, 1–946415. 23843684 10.1155/2013/946427 PMC3697395

[9] Choi KH, Joo BS, Sun ST, Park MJ, Son JB, Joo JK, Lee KS. Administration of visfatin during superovulation improves developmental competency of oocytes and fertility potential in aged female mice. Fertil Steril 2012; 97:1234–1241.e3. 22425197 10.1016/j.fertnstert.2012.02.032

[10] Mlyczyńska E, Zaobidna E, Rytelewska E, Dobrzyń K, Kieżun M, Kopij G, Szymańska K, Kurowska P, Dall'Aglio C, Smolińska N, Kamiński T, Rak A. Expression and regulation of visfatin/NAMPT in the porcine corpus luteum during the estrous cycle and early pregnancy. Anim Reprod Sci 2023; 250:107212. 36913896 10.1016/j.anireprosci.2023.107212

[11] Thakre A, Gupta M, Magar SP, Bahiram KB, Sardar VM, Korde JP, Bonde SW, Hyder I. Transcriptional and translational abundance of visfatin (NAMPT) in buffalo ovary during estrous cycle and its in vitro effect on steroidogenesis. Domest Anim Endocrinol 2021; 75:106583. 33249344 10.1016/j.domaniend.2020.106583

[12] Morgan SA, Bringolf JB, Seidel ER. Visfatin expression is elevated in normal human pregnancy. Peptides 2008; 29:1382–1389. 18524416 10.1016/j.peptides.2008.04.010

[13] Mazaki-Tovi S, Romero R, Kim SK, Vaisbuch E, Kusanovic JP, Erez O, Chaiworapongsa T, Gotsch F, Mittal P, Nhan-Chang CL, Than NG, Gomez R, et al. Could alterations in maternal plasma visfatin concentration participate in the phenotype definition of preeclampsia and SGA? J Matern Fetal Neonatal Med 2010; 23:857–868. 19900033 10.3109/14767050903301017 PMC3554253

[14] Birdir C, Fryze J, Frölich S, Schmidt M, Köninger A, Kimmig R, Schmidt B, Gellhaus A. Impact of maternal serum levels of Visfatin, AFP, PAPP-A, sFlt-1 and PlGF at 11–13 weeks gestation on small for gestational age births. J Matern Fetal Neonatal Med 2017; 30:629–634. 27124371 10.1080/14767058.2016.1182483

[15] Zulfıkaroglu E, Isman F, Payaslı A, Kılıc S, Kucur M, Danısman N. Plasma visfatin levels in preeclamptic and normal pregnancies. Arch Gynecol Obstet 2010; 281:995–998. 19639329 10.1007/s00404-009-1192-z

[16] Hu W, Wang Z, Wang H, Huang H, Dong M. Serum visfatin levels in late pregnancy and pre-eclampsia. Acta Obstet Gynecol Scand 2008; 87:413–418. 18382866 10.1080/00016340801976012

[17] Telejko B, Kuzmicki M, Zonenberg A, Szamatowicz J, Wawrusiewicz-Kurylonek N, Nikolajuk A, Kretowski A, Gorska M. Visfatin in gestational diabetes: serum level and mRNA expression in fat and placental tissue. Diabetes Res Clin Pract 2009; 84:68–75. 19185944 10.1016/j.diabres.2008.12.017

[18] Jiang YK, Deng HY, Qiao ZY, Gong FX. Visfatin level and gestational diabetes mellitus: a systematic review and meta-analysis. Arch Physiol Biochem 2021; 127:468–478. 33476191 10.1080/13813455.2021.1874997

[19] Ognjanovic S, Ku TL, Bryant-Greenwood GD. Pre–B-cell colony–enhancing factor is a secreted cytokine-like protein from the human amniotic epithelium. Am J Obstet Gynecol 2005; 193:273–282. 16021090 10.1016/j.ajog.2004.11.003 PMC1382169

[20] Kendal-Wright CE, Hubbard D, Bryant-Greenwood GD. Chronic stretching of amniotic epithelial cells increases pre-B cell Colony-enhancing factor (PBEF/Visfatin) expression and protects them from apoptosis. Placenta 2008; 29:255–265. 18272217 10.1016/j.placenta.2007.12.008

[21] Iciek R, Brazert M, Wender-Ozegowska E, Pietryga M, Brazert J. Low placental visfatin expression is related to impaired glycaemic control and fetal macrosomia in pregnancies complicated by type 1 diabetes. J Physiol Pharmacol 2018; 69:61–66. 29769421 10.26402/jpp.2018.1.06

[22] Katwa LC, Seidel ER. Visfatin in pregnancy: proposed mechanism of peptide delivery. Amino Acids 2009; 37:555–558. 18953631 10.1007/s00726-008-0194-7

[23] Dawid M, Kurowska P, Pawlicki P, Kotula-Balak M, Milewicz T, Dupont J, Rak A. Visfatin (NAMPT) expression in human placenta cells in normal and pathological conditions and its hormonal regulation in trophoblast JEG-3 cells. PloS One 2024; 19:e0310389. 39292698 10.1371/journal.pone.0310389 PMC11410215

[24] Lea RG . Proliferation, Differentiation and Apoptosis in Pregnancy and Cancer. In: Barnea ER, Jauniaux E, Schwartz PE (eds.), Cancer and Pregnancy, vol. 1, 1st ed. London: Springer London; 2001: 216–228.

[25] Burton GJ, Charnock-Jones DS, Jauniaux E. Regulation of vascular growth and function in the human placenta. Reproduction 2009; 138:895–902. 19470597 10.1530/REP-09-0092

[26] Sharp AN, Heazell AEP, Crocker IP, Mor G. Placental apoptosis in health and disease. Am J Reprod Immunol 2010; 64:159–169. 20367628 10.1111/j.1600-0897.2010.00837.x PMC3025811

[27] Chen CP, Bajoria R, Aplin JD. Decreased vascularization and cell proliferation in placentas of intrauterine growth–restricted fetuses with abnormal umbilical artery flow velocity waveforms. Am J Obstet Gynecol 2002; 187:764–769. 12237661 10.1067/mob.2002.125243

[28] Kaya B, Nayki U, Nayki C, Ulug P, Oner G, Gultekin E, Yildirim Y. Proliferation of trophoblasts and Ki67 expression in preeclampsia. Arch Gynecol Obstet 2015; 291:1041–1046. 25384521 10.1007/s00404-014-3538-4

[29] Magee TR, Ross MG, Wedekind L, Desai M, Kjos S, Belkacemi L. Gestational diabetes mellitus alters apoptotic and inflammatory gene expression of trophoblast from human term placenta. J Diabetes Complications 2014; 28:448–459. 24768206 10.1016/j.jdiacomp.2014.03.010 PMC4166519

[30] Carrasco-Wong I, Moller A, Giachini FR, Lima VV, Toledo F, Stojanova J, Sobrevia L, San MS. Placental structure in gestational diabetes mellitus. Biochim Biophys Acta 2020; 1866:165535. 10.1016/j.bbadis.2019.165535 31442531

[31] Orendi K, Gauster M, Moser G, Meiri H, Huppertz B. The choriocarcinoma cell line BeWo: syncytial fusion and expression of syncytium-specific proteins. Reproduction 2010; 140:759–766. 20696850 10.1530/REP-10-0221

[32] Zhuang BM, Cao DD, Liu XF, Wang L, Lin XL, Duan YG, Lee CL, Chiu PCN, Yeung WSB, Yao YQ. Application of a JEG-3 organoid model to study HLA-G function in the trophoblast. Front Immunol 2023; 14:1130308. 37006248 10.3389/fimmu.2023.1130308 PMC10050466

[33] Kar M, Ghosh D, Sengupta J. Histochemical and morphological examination of proliferation and apoptosis in human first trimester villous trophoblast. Hum Reprod 2007; 22:2814–2823. 17872910 10.1093/humrep/dem284

[34] Mlyczyńska E, Kurowska P, Drwal E, Opydo Chanek M, Tworzydło W, Kotula Balak M, Rak A. Apelin and apelin receptor in human placenta: expression, signalling pathway and regulation of trophoblast JEG 3 and BeWo cells proliferation and cell cycle. Int J Mol Med 2020; 45:691–702. 31922236 10.3892/ijmm.2020.4452 PMC7015120

[35] Mlyczyńska E, Myszka M, Kurowska P, Dawid M, Milewicz T, Bałajewicz-Nowak M, Kowalczyk P, Rak A. Anti-apoptotic effect of Apelin in human placenta: studies on BeWo cells and villous explants from third-trimester human pregnancy. Int J Mol Sci 2021; 22:2760. 33803239 10.3390/ijms22052760 PMC7967155

[36] Vega-Avila E, Pugsley MK. An overview of colorimetric assay methods used to assess survival or proliferation of mammalian cells. Proc West Pharmacol Soc 2011; 54:10–14. 22423572

[37] Livak KJ, Schmittgen TD. Analysis of relative gene expression data using real-time quantitative PCR and the 2−ΔΔCT method. Methods 2001; 25:402–408. 11846609 10.1006/meth.2001.1262

[38] Rak A, Drwal E, Wróbel A, Gregoraszczuk EŁ. Resistin is a survival factor for porcine ovarian follicular cells. Reproduction 2015; 150:343–355. 26159832 10.1530/REP-15-0255

[39] Salgame P, Varadhachary AS, Primiano LL, Fincke JE, Muller S, Monestier M. An ELISA for detection of apoptosis. Nucleic Acids Res 1997; 25:680–681. 9016614 10.1093/nar/25.3.680 PMC146463

[40] Cui XL, Brockman D, Campos B, Myatt L. Expression of NADPH oxidase isoform 1 (Nox1) in human placenta: involvement in preeclampsia. Placenta 2006; 27:422–431. 15993942 10.1016/j.placenta.2005.04.004 PMC2891430

[41] Li YX, Long DL, Liu J, Qiu D, Wang J, Cheng X, Yang X, Li RM, Wang G. Gestational diabetes mellitus in women increased the risk of neonatal infection via inflammation and autophagy in the placenta. Medicine 2020; 99:e22152. 33019392 10.1097/MD.0000000000022152 PMC7535644

[42] Tuschl G, Mueller S. Effects of cell culture conditions on primary rat hepatocytes—cell morphology and differential gene expression. Toxicology 2006; 218:205–215. 16337326 10.1016/j.tox.2005.10.017

[43] Koh YQ, Chan HW, Nitert MD, Vaswani K, Mitchell MD, Rice GE. Differential response to lipopolysaccharide by JEG-3 and BeWo human choriocarcinoma cell lines. Eur J Obstet Gynecol Reprod Biol 2014; 175:129–133. 24485668 10.1016/j.ejogrb.2013.12.032

[44] Spiller KL, Wrona EA, Romero-Torres S, Pallotta I, Graney PL, Witherel CE, Panicker LM, Feldman RA, Urbanska AM, Santambrogio L, Vunjak-Novakovic G, Freytes DO. Differential gene expression in human, murine, and cell line-derived macrophages upon polarization. Exp Cell Res 2016; 347:1–13. 26500109 10.1016/j.yexcr.2015.10.017

[45] Wiatrak B, Kubis-Kubiak A, Piwowar A, Barg E. PC12 cell line: cell types, coating of culture vessels, differentiation and other culture conditions. Cells 2020; 9:958. 32295099 10.3390/cells9040958 PMC7227003

[46] Burleigh DW, Kendziorski CM, Choi YJ, Grindle KM, Grendell RL, Magness RR, Golos TG. Microarray analysis of BeWo and JEG3 trophoblast cell lines: identification of differentially expressed transcripts. Placenta 2007; 28:383–389. 16797695 10.1016/j.placenta.2006.05.001

[47] Lin P, Fu J, Zhao B, Lin F, Zou H, Liu L, Zhu C, Wang H, Yu X. Fbxw8 is involved in the proliferation of human choriocarcinoma JEG-3 cells. Mol Biol Rep 2011; 38:1741–1747. 20878477 10.1007/s11033-010-0288-7

[48] Rothbauer M, Patel N, Gondola H, Siwetz M, Huppertz B, Ertl P. A comparative study of five physiological key parameters between four different human trophoblast-derived cell lines. Sci Rep 2017; 7:5892. 28724925 10.1038/s41598-017-06364-z PMC5517571

[49] Szabat M, Page MM, Panzhinskiy E, Skovsø S, Mojibian M, Fernandez-Tajes J, Bruin JE, Bround MJ, Lee JTC, Xu EE, Taghizadeh F, O’Dwyer S, et al. Reduced insulin production relieves endoplasmic reticulum stress and induces β cell proliferation. Cell Metab 2016; 23:179–193. 26626461 10.1016/j.cmet.2015.10.016

[50] Zhang X, Sheng X, Miao T, Yao K, Yao D. Effect of insulin on thyroid cell proliferation, tumor cell migration, and potentially related mechanisms. Endoc Res 2019; 44:55–70. 10.1080/07435800.2018.1522641 30260725

[51] Shahbazi M, Cundiff P, Zhou W, Lee P, Patel A, D’Souza SL, Abbasi F, Quertermous T, Knowles JW. The role of insulin as a key regulator of seeding, proliferation, and mRNA transcription of human pluripotent stem cells. Stem Cell Res Ther 2019; 10:228. 31358052 10.1186/s13287-019-1319-5 PMC6664730

[52] Silva C, Nunes C, Correia-Branco A, Araújo JR, Martel F. Insulin exhibits an Antiproliferative and hypertrophic effect in first trimester human Extravillous trophoblasts. Reprod Sci 2017; 24:582–594. 27662903 10.1177/1933719116667220

[53] Vega M, Mauro M, Williams Z. Direct toxicity of insulin on the human placenta and protection by metformin. Fertil Steril 2019; 111:489–496.e5. 30709546 10.1016/j.fertnstert.2018.11.032

[54] Lin TC . The role of visfatin in cancer proliferation, angiogenesis, metastasis, drug resistance and clinical prognosis. Cancer Manag Res 2019; 11:3481–3491. 31114381 10.2147/CMAR.S199597 PMC6497876

[55] Annie L, Gurusubramanian G, Roy VK. Inhibition of visfatin by FK866 mitigates pathogenesis of cystic ovary in letrozole-induced hyperandrogenised mice. Life Sci 2021; 276:119409. 33781825 10.1016/j.lfs.2021.119409

[56] Gurel D, Özer E, Altunyurt S, Guclu S, Demir N. Expression of IGR-IR and VEGF and trophoblastic proliferative activity in placentas from pregnancies complicated by IUGR. Pathol Res Pract 2003; 199:803–809. 14989492 10.1078/0344-0338-00499

[57] Unek G, Ozmen A, Kipmen-Korgun D, Korgun ET. Immunolocalization of PCNA, Ki67, p27 and p57 in normal and dexamethasone-induced intrauterine growth restriction placental development in rat. Acta Histochem 2012; 114:31–40. 21371741 10.1016/j.acthis.2011.02.002

[58] Genbacev O, Joslin R, Damsky CH, Polliotti BM, Fisher SJ. Hypoxia alters early gestation human cytotrophoblast differentiation/invasion in vitro and models the placental defects that occur in preeclampsia. J Clin Invest 1996; 97:540–550. 8567979 10.1172/JCI118447 PMC507049

[59] Bosco Becerra C, Díaz Guerra E, Gutierrez Rojas R, González Montero J, Parra Cordero M, Rodrigo Salinas R, Barja YP. Placental hypoxia developed during preeclampsia induces Telocytes apoptosis in chorionic villi affecting the maternal-Fetus metabolic exchange. Curr Stem Cell Res Ther 2016; 11:420–425. 25643124 10.2174/1574888x10666150202144855

[60] Unek G, Ozmen A, Mendilcioglu I, Simsek M, Korgun ET. The expression of cell cycle related proteins PCNA, Ki67, p27 and p57 in normal and preeclamptic human placentas. Tissue Cell 2014; 46:198–205. 24852133 10.1016/j.tice.2014.04.003

[61] Dubova EA, Pavlov KA, Yesayan RM, Nagovitsyna MN, Tkacheva ON, Shestakova MV, Shchegolev AI. Morphometric characteristics of placental villi in pregnant women with diabetes. Bull Exp Biol Med 2011; 151:650–654. 22462069 10.1007/s10517-011-1406-9

[62] Oğlak SC, Obut M. Expression of ADAMTS13 and PCNA in the placentas of gestational diabetic mothers. Int J Morphol 2021; 39:38–44.

[63] Szymanska K, Rytelewska E, Zaobidna E, Kiezun M, Gudelska M, Kopij G, Dobrzyn K, Mlyczynska E, Kurowska P, Kaminska B, Nynca A, Smolinska N, et al. The effect of Visfatin on the functioning of the porcine pituitary gland: an in vitro study. Cells 2023; 12:2835. 38132154 10.3390/cells12242835 PMC10742260

[64] Brown JEP, Onyango DJ, Ramanjaneya M, Conner AC, Patel ST, Dunmore SJ, Randeva HS. Visfatin regulates insulin secretion, insulin receptor signalling and mRNA expression of diabetes-related genes in mouse pancreatic β-cells. J Mol Endocrinol 2010; 44:171–178. 19906834 10.1677/JME-09-0071

[65] Wei BR, Xu C, Rote NS. Increased resistance to apoptosis during differentiation and syncytialization of BeWo choriocarcinoma cells. Adv Biosci Biotechnol 2012; 03:805–813. 10.4236/abb.2012.326100 PMC5881895 29623239

[66] Smith S, Baker PN, Symonds EM. Placental apoptosis in normal human pregnancy. Am J Obstet Gynecol 1997; 177:57–65. 9240583 10.1016/s0002-9378(97)70438-1

[67] Chen R, Kang R, Fan XG, Tang D. Release and activity of histone in diseases. Cell Death Dis 2014; 5:e1370. 25118930 10.1038/cddis.2014.337 PMC4454312

[68] Kiraz Y, Adan A, Kartal Yandim M, Baran Y. Major apoptotic mechanisms and genes involved in apoptosis. Tumour Biol 2016; 37:8471–8486. 27059734 10.1007/s13277-016-5035-9

[69] Zhang Z, Xiao K, Wang S, Ansari AR, Niu X, Yang W, Lu M, Yang Z, Rehman ZU, Zou W, Bei W, Song H. Visfatin is a multifaceted molecule that exerts regulation effects on inflammation and apoptosis in RAW264.7 cells and mice immune organs. Front Immunol 2022; 13:1018973. 36532047 10.3389/fimmu.2022.1018973 PMC9753570

[70] Annie L, Gurusubramanian G, Kumar RV. Visfatin protein may be responsible for suppression of proliferation and apoptosis in the infantile mice ovary. Cytokine 2021; 140:155422. 33476980 10.1016/j.cyto.2021.155422

[71] Tertemiz F, Kayisli UA, Arici A, Demir R. Apoptosis contributes to vascular lumen formation and vascular branching in human placental Vasculogenesis. Biol Reprod 2005; 72:727–735. 15564598 10.1095/biolreprod.104.034975

[72] Endo H, Okamoto A, Yamada K, Nikaido T, Tanaka T. Frequent apoptosis in placental villi from pregnancies complicated with intrauterine growth restriction and without maternal symptoms. Int J Mol Med 2005; 16:79–84. 15942681

[73] Travaglino A, Raffone A, Saccone G, Migliorini S, Maruotti GM, Esposito G, Mollo A, Martinelli P, Zullo F, D’Armiento M. Placental morphology, apoptosis, angiogenesis and epithelial mechanisms in early-onset preeclampsia. Eur J Obstet Gynecol Reprod Biol 2019; 234:200–206. 30721786 10.1016/j.ejogrb.2018.12.039

[74] Belkacemi L, Kjos S, Nelson DM, Desai M, Ross MG. Reduced apoptosis in term placentas from gestational diabetic pregnancies. J Dev Orig Health Dis 2013; 4:256–265. 25054844 10.1017/S2040174413000068

[75] Massimino M, Sciacca L, Parrinello NL, Scalisi NM, Belfiore A, Vigneri R, Vigneri P. Insulin receptor isoforms differently regulate cell proliferation and apoptosis in the ligand-occupied and unoccupied state. Int J Mol Sci 2021; 22:8729. 34445431 10.3390/ijms22168729 PMC8395753

[76] Li Y, Zhang Y, Dorweiler B, Cui D, Wang T, Woo CW, Brunkan CS, Wolberger C, Imai S, Tabas I. Extracellular Nampt promotes macrophage survival via a nonenzymatic Interleukin-6/STAT3 Signaling mechanism. J Biol Chem 2008; 283:34833–34843. 18945671 10.1074/jbc.M805866200 PMC2596403

[77] Pérez-Pérez A, Maymó J, Dueñas JL, Goberna R, Calvo JC, Varone C, Sánchez-Margalet V. Leptin prevents apoptosis of trophoblastic cells by activation of MAPK pathway. Arch Biochem Biophys 2008; 477:390–395. 18619412 10.1016/j.abb.2008.06.015

[78] Duval F, Santos ED, Poidatz D, Sérazin V, Gronier H, Vialard F, Dieudonné MN. Adiponectin inhibits nutrient transporters and promotes apoptosis in human villous Cytotrophoblasts: involvement in the control of Fetal growth. Biol Reprod 2016; 94:1–12. 10.1095/biolreprod.115.134544 27030046

[79] Dessì A, Marincola FC, Fanos V. Metabolomics and the great obstetrical syndromes – GDM, PET, and IUGR. Best Pract Res Clin Obstet Gynaecol 2015; 29:156–164. 25271062 10.1016/j.bpobgyn.2014.04.023

[80] Unek G, Ozmen A, Isenlik BS, Korgun ET. The proliferation mechanism of normal and pathological human placentas. Histol Histopathol 2017; 32:339–349. 27665761 10.14670/HH-11-832

[81] Dawid M, Pich K, Mlyczyńska E, Respekta-Długosz N, Wachowska D, Greggio A, Szkraba O, Kurowska P, Rak A. Adipokines in pregnancy. Adv Clin Chem 2024; 121:172–269. 38797542 10.1016/bs.acc.2024.04.006

[82] Miehle K, Stepan H, Fasshauer M. Leptin, adiponectin and other adipokines in gestational diabetes mellitus and pre-eclampsia. Clin Endocrinol (Oxf) 2012; 76:2–11. 21951069 10.1111/j.1365-2265.2011.04234.x

[83] Chen H, Chen H, Wu Y, Liu B, Li Z, Wang Z. Adiponectin exerts antiproliferative effect on human placenta via modulation of the JNK/c-Jun pathway. Int J Clin Exp Pathol 2014; 7:2894–2904. 25031708 PMC4097280

