# Supplementary material for: Visfatin exerts an anti-proliferative and pro-apoptotic effect in the human placenta cells
Source: Biol Reprod. 2024 Nov 19;112(2):375–91. doi: 10.1093/biolre/ioae168 (PMC11833490; doi:10.1093/biolre/ioae168)
Supplement: Supplementary_Table_1_ioae168 [file supplementary_table_1_ioae168.docx]

**Supplementary Table 1.** List of abbreviations used in the manuscript.

| Abbreviation | Explanation |
| --- | --- |
| AKT | Protein kinase B |
| AMPKα | 5′ adenosine monophosphate-activated protein kinase |
| BAX | Bcl-2-like protein 4 |
| BCL2 | B-cell lymphoma 2 |
| BrdU | Bromodeoxyuridine |
| BSA | Bovine serum albumin |
| CASP3 | Caspase-3 |
| CASP3/7 | Caspase-3/7 |
| CASP8 | Caspase-8 |
| CASP9 | Caspase-9 |
| CCNA2 | Cyclin A2 gene |
| CCNB1 | Cyclin B1 gene |
| CCND1 | Cyclin D1 gene |
| CCNE1 | Cyclin E1 gene |
| cDNA | Complementary DNA |
| DMEM/F12 | Dulbecco’s modified eagle medium/nutrient mixture f-12 |
| ELISA | Enzyme-linked immunosorbent assay |
| ERK1/2 | Extracellular signal-activated kinase |
| FBS | Fetal bovine serum |
| GAPDH | Glyceraldehyde 3-phosphate dehydrogenase |
| GDM | Gestational diabetes mellitus |
| HRP | Horseradish peroxidase |
| HSD | Honestly significant difference |
| IL-8 | Interleukin-8 |
| INS | Insulin |
| INSR | Insulin receptor |
| IUGR | Intrauterine growth restriction |
| mRNA | Messenger RNA |
| NAMPT | Nicotinamide phosphoribosyltransferase |
| P53 | Tumor protein 53 |
| PBS | Phosphate-buffered saline |
| PCNA | Proliferating cell nuclear antigen |
| PE | Preeclampsia |
| PI | Propidium iodide |
| PVDF | Polyvinylidene fluoride |
| RT-qPCR | Real-time polymerase chain reaction |
| STAT3 | Signal transducer and activator of transcription 3 |
