# Supplementary material for: Visfatin exerts an anti-proliferative and pro-apoptotic effect in the human placenta cells
Source: Biol Reprod. 2024 Nov 19;112(2):375–91. doi: 10.1093/biolre/ioae168 (PMC11833490; doi:10.1093/biolre/ioae168)
Supplement: Supplementary_Table_2_ioae168 [file supplementary_table_2_ioae168.docx]

**Supplementary Table 2.** Specifications of TaqMan’s used in the RT-qPCR analysis. *PCNA*- proliferating cell nuclear antigen, *CCND1*- cyclin D, *CCNE1*- cyclin E, *CCNA2*- cyclin A, *CCNB*- cyclin B1, ***P53*- tumor protein p53, *BAX*- bcl-2-like protein 4, *BCL2*- B-cell lymphoma 2, *CASP8*- caspase 8, *CASP9*- caspase 9, *CASP3*- caspase 3, *INSR*- insulin receptor, *GAPDH*- glyceraldehyde 3-phosphate dehydrogenase,** Thermo Fisher Scientific (Waltham, MA, USA)**.**

| Genes | Assay sequences | Reference sequences |
| --- | --- | --- |
| *PCNA* | Hs00696862_m1 | NM_002592.2 |
| *CCND1* | Hs00765553_m1 | NM_053056.2 |
| *CCNE1* | Hs01026536_m1 | NM_001238.3 |
| *CCNA2* | Hs00171105_m1 | NM_001111045.1 |
| *CCNB1* | Hs99999188_m1 | NM_031966.3 |
| *P53* | Hs01034249_m1 | NM_000546.5 |
| *BAX* | Hs00180269_m1 | NM_001291428.1 |
| *BCL2* | Hs04986394_s1 | NM_000633.2 |
| CASP8 | Hs06630780_s18 | NM_001080124.1 |
| *CASP9* | Hs00962278_m1 | NM_001229.4 |
| *CASP3* | Hs00234387_m1 | NM_004346.3 |
| *INSR* | Hs00961557_m1 | NM_000208.3 |
| *GAPDH* | Hs02786624_g1 | NM_001256799.2 |
