# Supplementary material for: Visfatin exerts an anti-proliferative and pro-apoptotic effect in the human placenta cells
Source: Biol Reprod. 2024 Nov 19;112(2):375–91. doi: 10.1093/biolre/ioae168 (PMC11833490; doi:10.1093/biolre/ioae168)
Supplement: Supplementary_Table_3_ioae168 [file supplementary_table_3_ioae168.docx]

**Supplementary Table 3.** Specifications of primary and secondary antibodies used in the Western blotting analysis. PCNA- proliferating cell nuclear antigen, **P53- tumor protein p53, BAX- bcl-2-like protein 4, BCL2- B-cell lymphoma 2, CASP8- caspase 8, CASP9- caspase 9, CASP3- caspase 3, ERK1/2- extracellular signal-activated kinase, AKT- protein kinase B, STAT3- signal transducer and activator of transcription 3, AMPKα- 5'AMP-activated kinase, INSR- insulin receptor, ACTB- β-actin, p- phosphorylated isoform, t- total isoform,** Thermo Fisher Scientific (Waltham, MA, USA), Cell Signaling (Danvers, MA, USA), Sigma-Aldrich (Saint Louis, MO, USA), Abcam (Cambridge, UK).

|  | Host species | Antibody types | Catalog numbers  and concerns | Research Resource Identifiers | Dilutions |
| --- | --- | --- | --- | --- | --- |
| PRIMARY ANTIBODIES | | | | | |
| anti-PCNA | mouse | monoclonal | no. 13-3900,  Thermo Fisher Scientific | AB_86593 | 1:500 |
| anti-CYCLIN D1 | rabbit | monoclonal | no. 2978T,  Cell Signaling | AB_2750906 | 1:500 |
| anti-CYCLIN E1 | mouse | monoclonal | no. ab392,  Abcam | AB_304167 | 1:1000 |
| anti-CYCLIN A2 | mouse | monoclonal | no. 4656T,  Cell Signaling | AB_304138 | 1:500 |
| anti-CYCLIN B1 | rabbit | monoclonal | no.4138T,  Cell Signaling | AB_731779 | 1:500 |
| anti-P53 | rabbit | polyclonal | no. 9282S,  Cell Signaling | AB_331476 | 1:500 |
| anti-BAX | rabbit | polyclonal | no. 2772S,  Cell Signalling | AB_10695870 | 1:1000 |
| anti-BCL2 | rabbit | monoclonal | no. 4223S,  Cell Signaling | AB_1903909 | 1:1000 |
| anti-CASP8 | mouse | monoclonal | no. MA1-41280,  Thermo Fisher Scientific | AB_1071016 | 1:1000 |
| anti-CASP9 | rabbit | monoclonal | no. 9502S,  Cell Signaling | AB_2068620 | 1:700 |
| anti-CASP3 | rabbit | polyclonal | no. 9662S,  Cell Signaling | AB_331439 | 1:1000 |
| anti-pERK1/2 | rabbit | polyclonal | no. 9101S,  Cell Signaling | AB_331646 | 1:1000 |
| anti-tERK1/2 | rabbit | polyclonal | no. 9102S,  Cell Signaling | AB_330744 | 1:1000 |
| anti-pAKT | rabbit | polyclonal | no. 9271T,  Cell Signaling | AB_329825 | 1:700 |
| anti-tAKT | rabbit | polyclonal | no. 9272S,  Cell Signaling | AB_329827 | 1:700 |
| anti-pSTAT3 | rabbit | polyclonal | no. 9131S,  Cell Signaling | AB_331586 | 1:500 |
| anti-tSTAT3 | rabbit | polyclonal | no. 9102S,  Cell Signaling | AB_331588 | 1:500 |
| anti-pAMPKα | rabbit | polyclonal | no. PA5-17831,  Thermo Fisher Scientific | AB_330330 | 1:500 |
| anti-tAMPKα | rabbit | polyclonal | no. PA5-17398,  Thermo Fisher Scientific | AB_330331 | 1:500 |
| anti-INSR | rabbit | polyclonal | no. BS-0681R,  Thermo Fisher Scientific | AB_10855934 | 1:700 |
| anti-visfatin | rabbit | polyclonal | no. ab233294,  Abcam | AB_3095753 | 1:50 |
| anti-ACTB | mouse | monoclonal | no. A5316,  Sigma-Aldrich | AB_476743 | 1:1000 |
| SECONDARY ANTIBODIES | | | | | |
| anti-rabbit | goat | polyclonal | no. 7074,  Cell Signaling | AB_2099233 | 1:1000 |
| anti-mouse | horse | polyclonal | no. 7076,  Cell Signaling | AB_330924 | 1:1000 |
| anti-rabbit  Alexa Fluor™ 488 | goat | polyclonal | no. A-11008,  Thermo Fisher Scientific | AB_143165 | 1500 |
| anti-rabbit  Alexa Fluor™ 594 | goat | polyclonal | no. A-11012,  Thermo Fisher Scientific | AB_2534079 | 1:500 |
